# A New Member of the Growing Family of Contact-Dependent Growth Inhibition Systems in *Xenorhabdus doucetiae*

**DOI:** 10.1371/journal.pone.0167443

**Published:** 2016-12-01

**Authors:** Jean-Claude Ogier, Bernard Duvic, Anne Lanois, Alain Givaudan, Sophie Gaudriault

**Affiliations:** DGIMI, INRA, Université de Montpellier, Montpellier, France; Centre National de la Recherche Scientifique, Aix-Marseille Université, FRANCE

## Abstract

*Xenorhabdus* is a bacterial symbiont of entomopathogenic *Steinernema* nematodes and is pathogenic for insects. Its life cycle involves a stage inside the insect cadaver, in which it competes for environmental resources with microorganisms from soil and the insect gut. *Xenorhabdus* is, thus, a useful model for identifying new interbacterial competition systems. For the first time, in an entomopathogenic bacterium, *Xenorhabdus doucetiae* strain FRM16, we identified a *cdi*-like locus. The *cdi* loci encode contact-dependent inhibition (CDI) systems composed of proteins from the two–partner secretion (TPS) family. CdiB is the outer membrane protein and CdiA is the toxic exoprotein. An immunity protein, CdiI, protects bacteria against inhibition. We describe here the growth inhibition effect of the toxic C-terminus of CdiA from *X*. *doucetiae* FRM16, CdiA-CT^FRM16^, following its production in closely and distantly related enterobacterial species. CdiA-CT^FRM16^ displayed Mg^2+^-dependent DNase activity, *in vitro*. CdiA-CT^FRM16^-mediated growth inhibition was specifically neutralized by CdiI^FRM16^. Moreover, the *cdi* ^FRM16^ locus encodes an ortholog of toxin-activating proteins C that we named CdiC^FRM16^. In addition to *E*. *coli*, the *cdiBCAI-*type locus was found to be widespread in environmental bacteria interacting with insects, plants, rhizospheres and soils. Phylogenetic tree comparisons for CdiB, CdiA and CdiC suggested that the genes encoding these proteins had co-evolved. By contrast, the considerable variability of CdiI protein sequences suggests that the *cdiI* gene is an independent evolutionary unit. These findings further characterize the sparsely described *cdiBCAI-*type locus.

## Introduction

*Xenorhabdus*, a member of the Enterobacteriaceae family, is a natural symbiont of entomopathogenic *Steinernema* spp. nematodes living in the soil [1, 2]. The bacterium-nematode symbiosis is used for the biological control of various groups of insects [3], but the genus *Xenorhabdus* is also a pertinent and tractable model for investigating processes of antagonism between bacteria [4]. Indeed, the complex lifecycle of this bacterium involves an alternation between ecological niches: the nematode gut, the hemocoel of the living insect and the insect cadaver. *Xenorhabdus* proliferation in the insect cadaver is dependent on the ability of the bacterium to kill other microorganisms living in the insect gut, on the nematode cuticle or in soils, which would otherwise compete for resources.

*Xenorhabdus* produces a broad diversity of bioactive secreted metabolites with antimicrobial activity [5]. For example, xenocoumacins [6–8], xenortides [9], xenematides [10] and PAX-peptides [11, 12], which target a broad spectrum of microorganisms, are synthesized by the non-ribosomal peptide synthetase (NRPS) enzymes and polyketide synthase (PKS) enzymes of *Xenorhabdus* strains. Antagonism with other *Xenorhabdus* or closely related bacterial genera may also be mediated by ribosomal-encoded bacteriocins [13, 14]. *Xenorhabdus* produces phage-derived bacteriocins [15, 16] and colicin E3-type killer proteins [17].

New bacterial antagonism systems requiring direct cell-to-cell contact for toxin delivery between Gram-negative bacteria have been described in the last decade. Bacterial type 6 secretion systems (T6SS) were first described in *Vibrio cholerae* [18]. In several bacterial taxa, the T6SS injects toxic effectors into competing bacteria to inhibit their growth [19–21]. *Xenorhabdus* genomes harbor several clusters of genes encoding putative T6SS [22, 23], but the functional role of the products of these genes has yet to be demonstrated. Contact-dependent growth inhibition (CDI) systems have been shown to mediate interbacterial competition in a contact-dependent manner. CDI involves a two-partner secretion (TPS) system, a member of the type 5 secretion system found in Gram-negative bacteria [24–26]. CDI systems are composed of two proteins encoded in a same genomic cluster: CdiB, an outer membrane β-barrel protein and CdiA, a large and secreted exoprotein. The growth inhibition activity resides within the carboxy-terminal region of CdiA of ~ 300 amino acids (CdiA-CT). The CDI systems display the typical features of the large growing family of polymorphic toxin systems: a protein, CdiI, encoded immediately downstream of the CdiA-encoding gene, protects CDI+ bacterial cells from growth inhibition, each CdiA-CT toxin is specifically neutralized by its cognate CdiI protein and the CdiA-CT toxin domains and the CdiI immunity proteins are highly polymorphic [27]. The CDI toxin/immunity complex therefore serves as a basis for self/non-self discrimination during inter-strain competition. Furthermore, it was recently shown to participate to the stabilization of mobile genetic elements in bacterial cells [28]. Clusters of genes encoding putative CDI systems are mainly found throughout the Proteobacteria [27, 29]. Two different *cdi* loci have been characterized functionally: the “*E*. *coli*-type”, which displays the typical *cdiBAI* organization and is widespread in the Enterobacteriaceae [27, 30] and in the genus *Pseudomonas* [31], and the “*Burkholderia*-type*”*, with its typical *bcpAIOB* organization [32, 33].

We describe here a functional *cdi* locus in the entomopathogenic bacterium *Xenorhadbus doucetiae* strain FRM16. This locus has an atypical gene organisation, *cdiBCAI*, and is widespread in Gram-negative bacteria interacting with insects or plants, or inhabiting soils.

## Materials and Methods

### Bacterial strains, plasmids and primers

All the bacterial strains used in this study are presented in S1 Table. Bacteria were routinely grown in Luria–Bertani (LB) medium at 28°C (*Xenorhabdus* strains) or 37°C (*E*. *coli* strains). Kanamycin (20 mg.L^-1^) and ampicillin (150 mg.L^-1^) were added to the medium as required. P_*tet*_ promoters were induced by adding anhydrotetracycline (aTc) at a final concentration of 0.1 mg. L^-1^. The primers (Eurogentec) and plasmids used in this study are described in S1 Table.

### Plasmid construction for toxicity assays

The CdiA-CT^FRM16^ fragment was amplified by PCR with the primer pairs F_EcorI_Cter_tpsA_III/ R_sal1_tpsA_III. The PCR product was digested with *Eco*RI and *Sal*I and inserted into the corresponding sites of pGJ907 to yield pGJ907_CdiA-CT^FRM16^. Constructs were checked by sequencing (MWG-Eurofins, Germany). Plasmids pGJ907 and pGJ907_ CdiA-CT^FRM16^ were introduced into *E*. *coli* strain EPI400 (Epicentre, France) or *E*. *coli* strain WM3064 by transformation. The WM3064 transformants were used to transfer plasmids pGJ907 and pGJ907_ CdiA-CT^FRM16^ inside *Xenorhabdus bovienii* strain CS03 by conjugative mating (according to the protocol described by [34]) to construct the recombinant strain Xb_CS03_ PGJ907 and Xb_CS03_ PGJ907_ CdiA-CT^FRM16^.

XDD1_1118 and XDD1_1120 were amplified by PCR with the primer pairs XD1118_EcoR1_F/ XD1118_sal1_R and XD1120_EcoR1_F/ XD1120_sal1_R, respectively. The PCR products were digested with *Eco*RI and *Sal*I and inserted into the corresponding sites of pUC18 to yield plasmids pUC18_*XD1118* and pUC18_*XD1120*. Competent *E*. *coli* EPI400 cells harbouring pGJ907_ CdiA-CT^FRM16^ (see above) were then transformed with pUC18_*XD1118 and* pUC18_*XD1120*, to yield the recombinant strains *E*. *coli*_ PGJ907_ CdiA-CT^FRM16^ / pUC18*_XD118* and *E*. *coli*_ PGJ907_ CdiA-CT^FRM16^ / pUC18_*XD1120*. As controls, plasmids pUC18 and pGJ907 were used to construct the recombinant strains *E*. *coli*_ PGJ907_ CdiA-CT^FRM16^ / pUC18 and *E*. *coli*_ PGJ907 / pUC18.

### Plasmid construction for protein overproduction in *E*. *coli* BL21

The *cdiA-CT*^*FRM16*^_XDD1_1120 fragment and the XDD1_1120 gene were amplified by PCR with the primer pairs F_EcorI_Cter_tpsA_III/ XD_1120_sal1R_bis and XD1120_EcoR1_F/ XD_1120_sal1R_bis, respectively. The PCR products were digested with *Eco*RI and *Sal*I and inserted into the corresponding sites of pET28b to yield pET28b_CdiA-CT^FRM16^_CdiI-His6 and pET28b_CdiI-His6.

Plasmids pET28b _CdiA-CT^FRM16^_CdiI-His6 and pET28b_CdiI-His6 were introduced into *E*. *coli* strain BL21 by transformation.

### Toxicity assays with CdiA-CT^FRM16^

*E*. *coli* strain EPI400 cells carrying pGJ907 (empty vector control), pGJ907_CdiA-CT^FRM16^ were cultured overnight in LB medium supplemented with kanamycin. The cultures were then diluted 1/500 in fresh LB supplemented with kanamycin and 200 μL of the resulting suspensions was used to inoculate 96-well plates (Greiner). The plates were incubated at 28°C, with orbital shaking, in an Infinite M200 microplate reader (Tecan). Absorbance at 600 nm was measured every 30 minutes. When the OD_600_ reached 0.15 to 0.2, aTc was (final concentration = 100 ng.mL^-1^) or was not (controls) added to the medium to induce CdiA-CT^FRM16^ expression under the control of the P_*tet*_ promoter. Growth was evaluated for ~10 hours. All experiments were performed in triplicate. We used the same protocol for *Xenorhabdus* growth inhibition assays.

### Modulation of growth inhibition

Recombinant EPI400 *E*. *coli*_ PGJ907_ CdiA-CT^FRM16^ / pUC18, *E*. *coli*_ PGJ907_ CdiA-CT^FRM16^ / pUC18*_XD118* and *E*. *coli*_ PGJ907_ CdiA-CT^FRM16^ / pUC18_*XD1120* cells were used to inoculate 96-well plates in LB medium supplemented with IPTG (0.2 mM final), and were incubated at 28°C, as described above. We added aTc to the medium when the OD_600_ reached 0.15 to 0.2, to induce CdiA-CT^FRM16^ expression under the control of the P_*tet*_ promoter. Growth was assessed for ~10 hours. *E*. *coli* strain EPI400 cells carrying pGJ907 and pUC18 were used as empty vectors control. All experiments were performed in triplicate.

### Protein purification

The purified CdiA-CT^FRM16^ was obtained from *E*. *coli* strain BL21 carrying pET28b_*cdiA*-CT^FRM16^_*cdiI*^FRM16^. CdiA-CT^FRM16^/CdiI^FRM16^-His6 complexes were first overproduced and purified under non denaturing conditions in reaction buffer (20 mM sodium phosphate pH 7.0, 150 mM NaCl, 10 mM β-mercaptoethanol) as previously described [27]. The complex was denatured in reaction buffer containing 6 M guanidine-HCl, and the CdiA-CT^FRM16^ protein was isolated from CdiI^FRM16^-His6 by Ni^2+^-affinity chromatography. Purified CdiA-CT^FRM16^ and CdiI^FRM16^-His6 proteins were refolded by dialysis against reaction buffer.

As a control, purified CdiI^FRM16^-His6 alone was obtained from *E*. *coli* strain BL21 carrying pET28b_cdiI^FRM16^ and purified by Ni^2+^-affinity chromatography, as described above (without the denaturation step).

Purified proteins were analyzed by SDS-PAGE on a 15% polyacrylamide gel in the presence of SDS as previously described [35]. The samples, 5 μg of purified fraction, were run under reducing conditions, reduction being achieved by treating (3 min, 100°C) the samples with a solution containing β-2-mercaptoethanol (0.5% final concentration). The molecular mass was determined by the use of Page Ruler^TM^ plus prestained protein ladder (Thermo Scientific, USA). Gels were stained with Coomassie brilliant blue.

### Pull down experiment

Purified CdiA-CT^FRM16^ (5 μM) was incubated with CdiI^FRM16^-His6 (5 μM) for 30 min at room temperature and an aliquot was removed for analysis by SDS-PAGE. 500 μL of Ni^2+^-NTA resin, previously washed four times with binding buffer (20 mM sodium phosphate pH 7.0 containing 150 mM sodium chloride and 10 mM β-mercaptoethanol), was then added and incubated for 1 h 30 at 4°C. The resin was then collected by centrifugation and the supernatant (unbound proteins) was collected for analysis by SDS-PAGE. Ni^2+^-NTA was washed with binding buffer three times, and the bound proteins were eluted in elution buffer (20 mM sodium phosphate pH 7.0 containing 150 mM sodium chloride, 10 mM β-mercaptoethanol and 250 mM imidazole). Samples were finally analysed by SDS-PAGE and the gel was stained with Coomassie blue as described above.

### Nuclease assays

The activity of purified CdiA-CT^FRM16^ was assayed *in vitro* on genomic DNA from Xd_FRM16. CdiA-CT^FRM16^ (final concentration of 1 μM) was incubated with 2 μg of DNA in 50 μL of sterilized water supplemented with 2 mM MgCl_2_ or CaCl_2_ for 1 h at 37°C. Where indicated, purified CdiI^FRM16^-His6 protein from pET28b_*cdiI*^*FRM16*^ (immunity protein purified alone without CdiA-CT) was included at different final concentrations and allowed to bind CdiA-CT^FRM16^ for 30 minutes at room temperature before the addition of substrate DNA. Reactions were quenched with EDTA, and the reaction mixture was subjected to electrophoresis in a 1% agarose gel stained with ethidium bromide.

### RNA isolation and RT-PCR analysis

*X*. *doucetiae* FRM16 in LB broth was incubated at 28°C (100 mL) with horizontal shaking. Three samples were collected at different incubation times: i) early exponential growth phase (OD at 600 nm = 0.75); ii) end of the exponential growth phase (OD at 600 nm = 1.70); iii) stationary phase (OD at 600 nm = 3.70).

At each sampling time, total RNA was extracted with the RNeasy Protect Bacteria miniprep kit (from Qiagen), including incubation with DNase I, according to the manufacturer’s instructions. RNA concentration was determined by measuring absorbance at 260 nm. For each RNA preparation, we assessed DNA contamination by carrying out a control PCR targeting the 16S ribosomal RNA gene, by using *Xenorhabdus*-specific 16S primers (see S1 Table). The quantity and quality of total and messenger RNA, respectively, were assessed with a NanoDrop 2000 spectrophotometer (Thermo Scientific) and an Agilent 2100 Bioanalyzer with the RNA 6000 Nano LabChip kit (Agilent). Total RNA (2 μg) was reverse-transcribed with Super Script IV Reverse Transcriptase (Invitrogen) and random hexamers (100 ng.μL^−1^, Applied Biosystems) according to the manufacturer’s instructions. The primers used to amplify *cdi* genes and *cdi* gene junctions are listed in S1 Table. All PCRs on cDNA were performed with *GoTaq* DNA polymerase (Promega) or *iProof* DNA Polymerase (BioRad), in accordance with the manufacturer’s recommendations. The final PCR products were subjected to electrophoresis in 1% agarose gels in TAE, alongside the 1 kb DNA Ladder Plus (Euromedex).

### Genomic analysis

We used the Genoscope microscopy platform (http://www.genoscope.cns.fr/agc/microscope/home/) to identify the *tpsAB* loci present in available *Xenorhabdus* and *Photorhabdus* complete genome sequences, by searching for protein sequences with the conserved NPNGI and NPNL motifs; the N-terminal TPS domain is a hallmark of TpsA proteins.

We used BlastP with default parameters to search for orthologs of the CdiB/CdiC/CdiA/CdiI proteins in other bacteria present in the NCBI non-redundant protein database.

### Phylogenetic analysis

A sequence alignment (Muscle) was generated and phylogenetic analysis methods (maximum-likelihood analysis) were used with the Seaview Platform (http://doua.prabi.fr/software/seaview), as described elsewhere [23].

The strains used in phylogenetic analysis and their accession numbers are shown in S2 Table.

## Results

### Xd FRM16 harbors a new *cdi* locus

**Fig 1:**
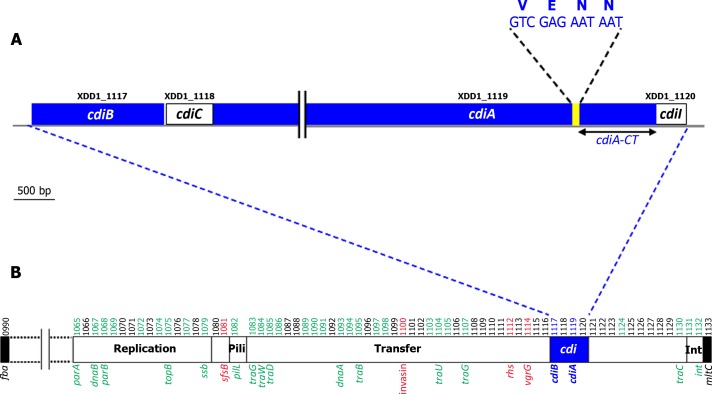
**The *cdiBCAI* locus of *Xenorhabdus doucetiae* FRM16 A**. **Genetic organization of the *cdiBCAI* locus** Boxes represent genes. Gene labels are shown above the boxes. The putative *cdiA* and *cdiB* genes are shown in blue. The *cdiA*-CT region is indicated. The location of the nucleotide sequence encoding the VENN motif is indicated in yellow. **B. The *cdiBCAI* locus is located in an integrative conjugative element (ICE).** The *cdi* locus is shown in blue. The conserved genes of the ICE, as defined in a previous study [40], are highlighted in green. Notable cargo genes (e.g. potentially involved in host interactions) are highlighted in red. The label numbers of the genes are indicated above the locus. The ICE is embedded in a large genomic island inserted between the *fba* and *mltC* genes (black boxes). The genetic content of the genomic island is described in S3 Table.

A putative *cdi* locus was identified in the genome of *Xenorhabdus doucetiae* strain FRM16 (Xd_FRM16). It contains four ORFs, XDD1_1117 to XDD1_1120 (Fig 1A). XDD1*_*1117 encodes a homolog of the outer membrane protein CdiB (29% and 36% identity to the CdiB of *E*. *coli* 536 and *Burkholderia thailandensis* E264, respectively). XDD1_1118 encodes a homolog of acyltransferase proteins generally described as toxin-activating protein C (34% identity to HlyC of *E*. *coli* strain PM152 [36]). For this reason, we named the ORF XDD1_1118 CdiC^FRM16^. XDD1_1119 is similar to CdiA (36% and 40% identity to the sequences of CdiA of *E*. *coli* 536 and *B*. *thailandensis* E264, respectively) and encodes the VENN motif separating the conserved N-terminus (~ 4 100 aa) from the variable C-terminus (~ 300 aa) in many CdiA proteins (Fig 1A). This carboxy-terminal region is referred to below as CdiA-CT^FRM16^. Finally, XDD1_1120 encodes a putative protein of unknown function. Its location just after the *cdiA* gene suggests that it may encode the CdiI immunity protein.

The *cdi* locus of Xd_FRM16 is located within a previously described integrative and conjugative element (ICE) [23] (Fig 1B) harbouring the remnant of a pilus synthesis locus (Fig 1B), but it contains the essential machinery for mobilization [23]. The ICE is embedded in a large genomic island (GI) inserted between the *fba* and *mltC* genes of the core genome (Fig 1B and S3 Table for genetic content). This GI contains several genes or gene clusters potentially involved in host interactions: i) the *mcf* gene, which encodes a toxin active against caterpillars [37], ii) a *paa*-like locus encoding enzymes of the phenylacetic acid catabolic pathway required for the oral pathogenicity of *Burkholderia cenocepacia* in *Caenorhabditis elegans* [38], iii) an *iol-*like locus encoding enzymes involved in myoinositol catabolism, the products of which have been implicated in the pathogenicity of the human fungal pathogen *Cryptococcus neoformans* [39].

### Growth inhibition function of the CdiA-CT^FRM16^ fragment and identification of the CdiI protein

**Fig 2:**
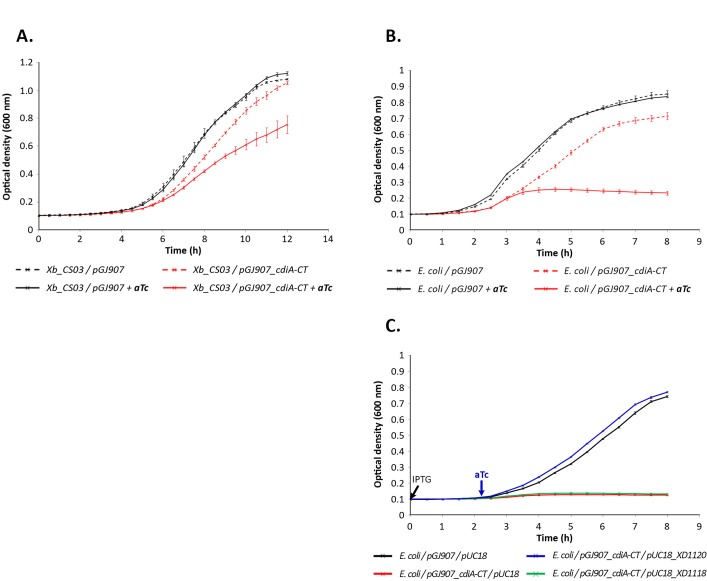
CdiA-CT^FRM16^ inhibits cell growth when expressed in *E*. *coli* and *Xenorhabdus bovienii*. **A. CdiA-CT**^**FRM16**^**–mediated growth inhibition in *X*. *bovienii* strain CS03 growth.** *X*. *bovienii* CS03 carrying pGJ907 (black lines), or pGJ907_*cdiA*-CT^FRM16^ (red lines) was grown at 28°C in LB broth supplemented with kanamycin (dotted curves), or LB broth supplemented with kanamycin plus aTc (continuous curves). **B. CdiA-CT**^**FRM16**^**–mediated growth inhibition in *E*. *coli* EPI400.** *E*. *coli* EPI400 carrying pGJ907 (black lines) or pGJ907_*cdiA*-CT^FRM16^ (red lines) was grown at 37°C in LB broth supplemented with kanamycin (dotted curves), or LB broth supplemented with kanamycin plus aTc (continuous curves). **C. XDD1-1120 confers immunity to CdiA-CT**^**FRM16**^ **toxicity in *E*. *coli* EPI400.** *E*. *coli* carrying pGJ907 and pUC18 (black line), pGJ907_*cdiA*-CT^FRM16^ and pUC18 (red line), pGJ907_*cdiA*-CT^FRM16^ and pUC18_ XD1120 (blue line), pGJ907_*cdiA*-CT^FRM16^ and pUC18_ XD1118 (green line) were grown at 37°C in LB broth supplemented with kanamycin. The times when IPTG and aTc were added at the culture are indicated by an arrow. In each panel, optical density at 600 nm was recorded every 30 minutes. When required, aTc was added 2 hours after the start of culture (OD_600 nm_~0.15). The results shown are the mean and standard deviation of three experiments.

The region encoding the CdiA-CT^FRM16^ polypeptide (residues 4167 to 4436) was inserted into plasmid pGJ907 under the control of the P_*tet*_ promoter, which is inducible by adding aTc (anhydrotetracycline) to the culture medium [41]. When introduced into *Xenorhabdus bovienii* strain CS03, which does not possess the *cdi* locus [42], the resulting plasmid, pGJ907_*cdiA*-CT^FRM16^, conferred a growth inhibition phenotype to the bacterium cultured in LB broth, upon aTc induction (Fig 2A). Similar results were obtained with *Escherichia coli* EPI400 (Fig 2B). The CdiA-CT^FRM16^ fragment therefore confers a capacity to inhibit the growth of both closely related (*X*. *bovienii*) and more distantly related (*E*. *coli*) species.

This growth inhibition was completely blocked in *E*. *coli*/pGJ907_*cdiA*-CT^FRM16^ following co-expression of the XDD1_1120 gene within pUC18, under the control of the P_*lac*_ promoter (Fig 2C). These results confirm that XDD1_1120 confers immunity to CdiA-CT^FRM16^ mediated growth inhibition. We therefore named the XDD1_1120-encoded protein CdiI^FRM16^. By contrast, the expression of *cdiC*^FRM16^gene (XDD1_1118) does not display significant consequence on growth inhibition caused by *cdiA*-CT^FRM16^ (Fig 2C).

### CdiA-CT^FRM16^ displays DNAse activity

**Fig 3:**
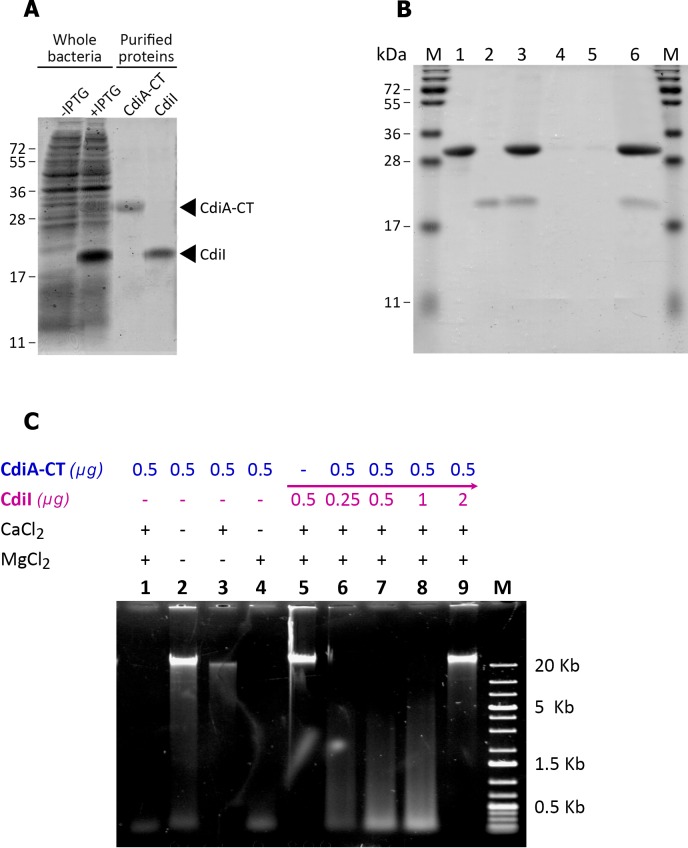
***In vitro* analysis of CdiA-CT**^**FRM16**^**/CdiI**^**FRM16**^ **activities A. SDS-polyacrylamide gel electrophoresis of the purified recombinant CdiA-CT**^**FRM16**^ **and CdiI**^**FRM16**^ **proteins.** Electrophoretic separation, by SDS-PAGE (15% acrylamide), of proteins after the culture in LB broth of *E*. *coli*/pET28b_ *cdiA*-CT^FRM16^_ XD1120 with or without IPTG induction (whole bacteria), or after purification by Ni^2+^-affinity chromatography (purified proteins). Proteins were visualized by Coomassie Blue staining. CdiA-CT^FRM16^ and CdiI^FRM16^ had apparent sizes of 30 kDa and 18 kDa, respectively. **B. CdiI**^**FRM16**^ **binds specifically to its cognate CdiA-CT.** Purified CdiA-CT^FRM16^ (**lane 1**) and CdiI^FRM16-^His6 purified alone (**lane 2**) were incubated in equimolar mixture (**lane 3**), and subjected to Ni^2+^-affinity chromatography to bind the CdiA-CT^FRM16^-CdiI ^FRM16^-His6 complex. The complex was washed twice to liberate the unbound protein fractions (**lanes 4 and 5**, respectively). The bound protein fraction (**lane 6**) was eluted using imidazole. All fractions were run on SDS-polyacrylamide gels and stained with Coomassie blue. **M**, protein marker (PageRuler plus prestained protein ladder, Thermo scientific). **C. CdiA-CT**^**FRM16**^ **has DNAse activity** CdiA-CT^FRM16^ was assayed for DNase activity with bacterial genomic DNA from Xd_FRM16. DNA was visualized by electrophoresis in a 0.7% agarose gel. ***Lanes 1 to 4***: The purified CdiA-CT^FRM16^ protein and genomic DNA were incubated for 1 hour at 37°C with or without CaCl_2_ (2 mM) and MgCl_2_ (2 mM). ***Lane 5*:** The CdiI^FRM16^ protein purified alone and genomic DNA were incubated for 1 hour at 37°C with CaCl_2_ (2 mM) and MgCl_2_ (2 mM). ***Lanes 6 to 9*:** The purified CdiA-CT^FRM16^ and CdiI^FRM16^ proteins were first incubated for 30 minutes at 37°C in the presence of various concentrations of CdiI^FRM16^. Genomic DNA was then added and the mixture was incubated for 1 hour at 37°C with CaCl_2_ (2 mM) and MgCl_2_ (2 mM). ***Lane M*:** 1-kb DNA ladder (Eurogentec).

The most closely related ortholog of CdiA-CT^FRM16^ is CdiA-CT from *Dickeya dadantii* 3937 (67% amino-acid identity), an Mg^2+^-dependent DNase [27]. We assessed the DNase activity of CdiA-CT^FRM16^, by using the pET28b vector to overproduce (i) the CdiA-CT^FRM16^ fragment and the immunity protein CdiI^FRM16^ (Fig 3A) or (ii) the immunity protein CdiI^FRM16^ alone. His6-tagged CdiI^FRM16^ was purified by Ni-NTA chromatography under denaturing conditions. The CdiA-CT^FRM16^ toxin was purified together with its cognate His6-tagged CdiI^FRM16^ immunity protein, to prevent autotoxicity in the *E*. *coli* strain overproducing it. It was then separated from the immunity protein by SDS-PAGE under denaturing conditions. The purified CdiA-CT^FRM16^ and CdiI^FRM16^ polypeptides had observed sizes of 30 and 18 kDa, respectively (Fig 3A), consistent with the predicted sizes of the recombinant products. Purified recombinant protein yield was ~500 mg.L^-1^ for CdiA-CT^FRM16^ and ~100 mg.L^-1^ for CdiI^FRM16^. We underwent pull-down experiment to confirm that the over-produced CdiI^FRM16^ immunity protein purified alone binds specifically to cognate over-produced CdiA-CT^FRM16^ protein (Fig 3B).

We then assessed the nuclease activity of the purified CdiA-CT^FRM16^ fragment *in vitro* (Fig 3C). Xd_FRM16 genomic DNA was completely degraded following incubation with 0.5 μg of purified CdiA-CT^FRM16^, in the presence of the bivalent cations Mg^2+^ and Ca^2+^, or Mg^2+^ alone (lanes 1 and 4, Fig 3C). CdiA-CT^FRM16^ also displayed DNase activity against genomic DNA from different *Xenorhabdu*s and *Photorhabdus* species, supercoiled plasmid DNA and eukaryotic genomic DNA (data not shown). In the absence of Mg^2+^ ions, little or no nuclease activity was observed in the absence or presence of Ca^2+^ cations (lanes 2 and 3, Fig 3C). The CdiI^FRM16^ polypeptide had no DNase activity (lane 5, Fig 3C). We then evaluated the impact on CdiA-CT^FRM16^ DNase activity of increasing concentrations of CdiI^FRM16^ by pre-incubation of the two polypeptides. Protection against DNase activity was observed for a CdiA-CT^FRM16^ / CdiI^FRM16^ ratio greater than two (lanes 6 to lanes 9, Fig 3C). Thus, CdiA-CT^FRM16^ displays Mg^2+^-dependent DNase activity that is neutralized by its cognate CdiI ^FRM16^ immunity protein.

### *cdiB*, *cdiC*, *cdiA* and *cdiI* belong to the same transcriptional unit

**Fig 4:**
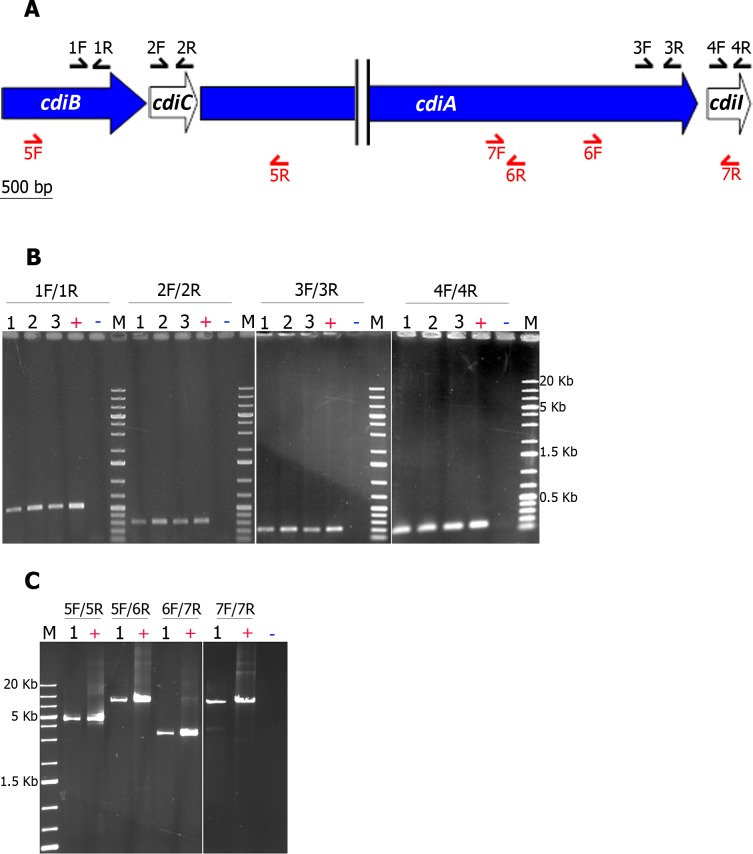
*Xd*_FRM16 *cdiC*, *cdiB* and *cdiA* are transcribed as a single transcription unit. **A. Position of the primers used in RT-PCR analyses.** The *cdi* genes are indicated by large arrows, the size of which is proportional to gene length. The positions of the primers are indicated by thin black arrows for internal PCRs and thin red arrows for junction PCRs (see S1 Table for a description of the primers). **B. *cdiB*, *cdiC*, *cdiA* and *cdiI* were transcribed at different time points during bacterial growth in LB broth.** cDNAs were synthesized from total RNA extracted from cultures of *X*. *doucetiae* FRM16 in LB broth at different incubation times: **1** (OD at 600 nm = 0.75), **2** (OD at 600 nm = 1.70), **3** (OD at 600 nm = 3.70). 50 ng of cDNA were then amplified with specific primers targeting the four genes of the *X*. *doucetiae* FRM16 *cdi* locus (see panel A and S1 Table). ***+***, ~70 ng of genomic DNA from *X*. *doucetiae* FRM16; ***-*,** 1 μL of water; **M**, 5 μL of 1 kb DNA Ladder Plus (Euromedex). The PCR products were subjected to electrophoresis in 1% agarose gels. **C. *cdiB*, *cdiC*, *cdiA* and *cdiI* genes form a single operon.** Specific primers allowing the mapping of the *cdiB-cdiC-cdiA* and the *cdiA-cdiI* junctions were used (see panel A and S1 Table). ~50 ng of cDNA of the condition **1** (panel B) were used. ***+***, ~70 ng of genomic DNA from *X*. *doucetiae* FRM16; ***-*,** 1 μL of water; **M**, 5 μL of 1 kb DNA Ladder Plus (Euromedex).

We investigated whether the *cdi* locus could be considered to function as an operon, by mapping the RNAs of the *cdi* genes by PCR (see Fig 4A for location of the primers). We checked that RNA is free of DNA by absence of 16S rDNA gene amplification (data not shown). We then showed that the four genes were transcribed at different time points during bacterial growth in LB (Fig 4B). We finally mapped the *cdi*^*FRM16*^ cDNA with primers allowing the amplification of nucleotides 400 of *cdiB* through 2779 of *cdiA* (4641-bp fragment flanked by the primers 5F and 5R) or of nucleotides 400 of *cdiB* through 6568 of *cdiA* (8430-bp fragment flanked by the primers 5F and 6R), and the amplification of nucleotides 10184 of *cdiA* through 287 of *cdiI* (3430-bp fragment flanked by the primers 6F and 7R) or of nucleotides 5625 of *cdiA* through 287 of *cdiI* (7988-bp fragment flanked by the primers 7F and 7R) (Fig 4C). The *cdiB-cdiC-cdiA* and *cdiA-cdiI* genes were respectively co-transcribed, consistent with an operon structure for the *cdi*^*FRM16*^ locus.

### The *cdiBCAI* locus is widespread in environmental bacteria interacting with insects, plants, rhizospheres and soils

We investigated the frequency of occurrence of the *cdiBCAI*-type locus in entomopathogenic bacteria, by searching for orthologs of genes encoding the CdiA, CdiB, CdiC and CdiI proteins in 41 *Xenorhabdus* and *Photorhabdus* available genomes. *cdi-*like loci are frequently redundant in all *Photorhabdus* genomes. However, a *cdiBCAI*-type locus was detected only in the *P*. *luminescens* BA1 genome. By contrast, *cdi-*like loci are present in only ~30% of *Xenorhabdus* genomes, but a majority of *Xenorhabdus cdi-*like loci were of *cdiBCAI* type. The *cdiBCAI*-type loci encountered in the genomes of *Xenorhabdus* and *P*. *luminescens* BA1 are described in Table 1.

**Table 1:** Inventory of *cdiBCAI*-type loci in *Xenorhabdus* and *Photorhabdus* genomes.

Organism	Putative CdiB proteins		Putative CdiC proteins		Putative CdiA proteins			Putative CdiI proteins	
	Label	Length (aa)	Label	Length (aa)	Label	Length (aa)	Motifs and domains	Label	Length (aa)
*X*. *doucetiae* FRM16^1^	XDD1_1117	567	XDD1_1118	179	XDD1_1119	4436	Haem-FhaB-VENN	XDD1_1120	153
*X*. *bovienii* SS-2004^2^	XBJ1_1975	453	XBJ1_1976	179	XBJ1_1977^***a***^/XBJ1_1979^***a***^	642/2663	FhaB-DUF638-VENN	Absent	/
*X*. *szentirmaii* DSM16338^3^	/	/	Absent	/	XSR1v1_770001	2539	Haem-FhaB-VENN	ND	/
	XSR1v1_900001	565	XSR1v1_900002	180	XSR1v1_900003^***a***^	438	/	ND	/
*X*. *cabanillasii* JM26^4^	XCR1v1_1000008	412	XCR1v1_1000009	179	XCR1v1_1000010^***a***^	779	Haem-FhaB-VENN	ND	/
	XCR1v1_1970003	488	XCR1v1_1970002	179	/	/	/	ND	/
					XCR1v1_1510010	3226	Haem-FhaB-VENN	ND	/
*Xenorhabdus sp*. GDc328^5^	LGYQ01_v1_1280018	565	LGYQ01_v1_1280017	179	LGYQ01_v1_1280016	4428	Haem-FhaB-IESN	ND	/
*X*. *griffiniae* BMMCBg^6^	LDNM01_v1_250020	565	LDNM01_v1_250019	179	LDNM01_v1_250018	4428	Haem-FhaB-IESN	LDNM01_v1_250017	74
*Xenorhabdus sp*. NBAII XenSa04^7^	JTHK01_v1_820003	566	JTHK01_v1_820004	179	JTHK01_v1_820005^***a***^	2608	Haem	ND	/
*P*. *luminescens* BA1^8^	JFGV01_v1_310043	570	JFGV01_v1_310044	179	JFGV01_v1_310045^***a***^	3216	Haem-FhaB	ND	/

^a^ The genes are fragmented (pseudogenes)

Accession numbers

^1^NZ_FO704550.1 and NZ_FO704549.1 (pl.)

^2^FN667741

^3^NZ_CBXF010000001.1

^4^NZ_CBXE000000000.1

^5^ LGYQ0000000

^6^ LDNM01000000

^7^NZ_JTHK00000000.1

^8^PRJNA217861

We then expanded our biodiversity survey by searching for proteins orthologous to CdiC^FRM16^, the hallmark of the *cdiBCAI*-type locus, in the NCBI public database. CdiC^FRM16^ orthologs are systematically encoded inside *cdi*-like loci (S4 Table). Most of the CdiC orthologs were associated with (i) entomopathogenic bacteria, such as *Xenorhabdus* and *Serratia*, (ii) bacteria interacting with plants, such as *Pseudomonas syringae* and *D*. *dadantii* or (iii) soil and rhizosphere bacteria, such as *Pseudomonas fluorescens*, iv) and numerous pathogenic enterobacteria, e.g. *E*. *coli* strains.

**Fig 5:**
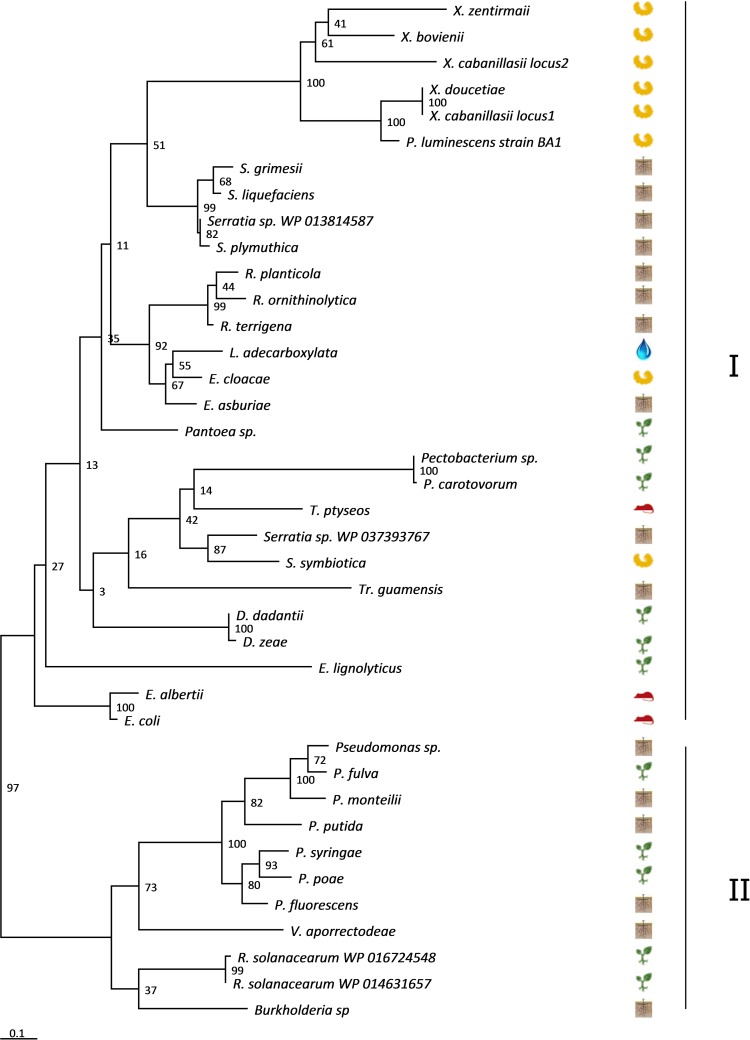
Phylogenetic analysis of CdiC ^FRM16^ orthologous sequences Phylogenetic trees were constructed by the maximum likelihood (ML) method, with bootstrap values indicated at the nodes. The branch length scale bar below the phylogenetic tree reflects the numbers of amino-acid substitutions per site. The protein sequences are split into two clades, I and II, indicated at the right of the tree. The ecological niches are symbolized by pictograms (insects, rhizosphere, water, plants, and vertebrates). Accession numbers of the sequences are indicated in S2 Table.

Phylogenetic analysis of a representative set of bacterial species clustered the CdiC sequences in two clades (Fig 5). Clade I contained CdiC sequences from Enterobacteriaceae, including environmental strains such as insects pathogens (*Xenorhabdus* strains, *P*. *luminescens* strain BA1 and *Serratia*) and plant pathogens (*Dickeya*, *Pantoea* and *Pectobacterium*), whereas clade II contained CdiC sequences from environmental proteobacteria of the Pseudomonadaceae and Burkholderiaceae families. Some features suggested probable horizontal genetic transfers of the *cdiC* gene between closely related species. For example, the CdiC *P*. *luminescens* BA1 sequence clustered with CdiC^FRM16^ (Fig 5). Moreover, the CdiC proteins of *X*. *doucetiae* and *X*. *cabanillasii* (locus 1) were 100% identical, despite the distant relationship of these two species within the genus *Xenorhabdus*.

### Evolutionary history of *cdiBCAI* genes

**Fig 6:**
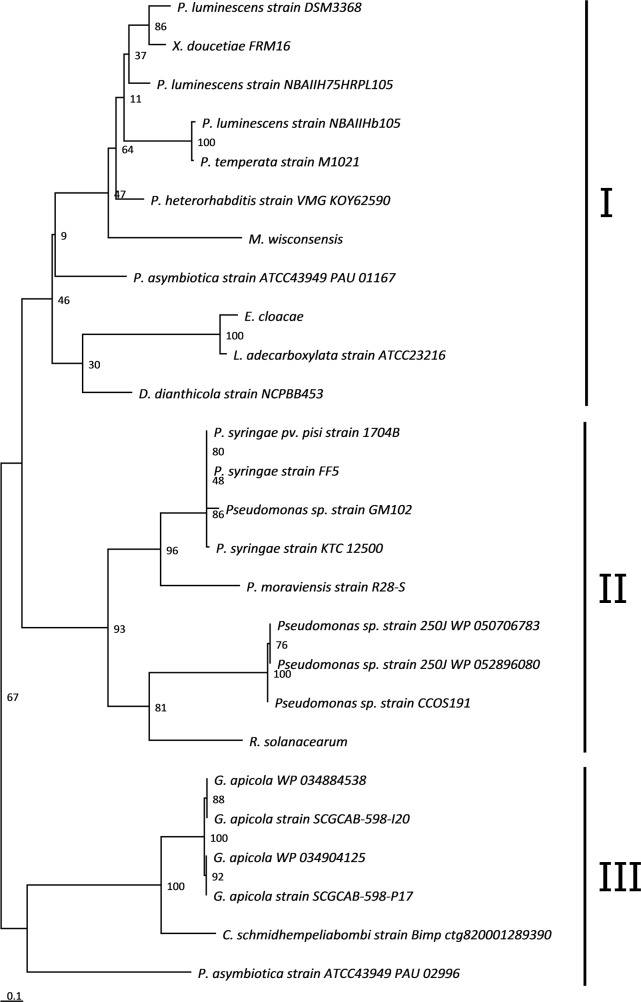
Phylogenetic analysis of CdiI^FRM16^ orthologous sequences Phylogenetic trees were constructed by the maximum likelihood (ML) method, with bootstrap values indicated at the nodes. The branch length scale bar below the phylogenetic tree reflects the numbers of amino-acid substitutions per site. The protein sequences are split into three clades, I, II and III, indicated at the right of the tree. Accession numbers of the sequences are indicated in S2 Table.

We analyzed the co-evolution of the Cdi proteins by comparing their phylogeny. As previously described for CdiC, the sequences of the CdiB, CdiA and CdiI proteins were retrieved by BlastP searches of the NCBI public database with CdiA^FRM16^, CdiB^FRM16^ and CdiI^FRM16^ as queries (S4 Table). The phylogenetic trees for the CdiB, CdiA and CdiC proteins were congruent, suggesting co-evolution of the three genes (S1 Fig). The CdiB and CdiA sequences from “*E*. *coli*-type” and “*Burkholderia*-type” *cdi* loci, used as references, branched outside these trees, confirming that the *cdiBCAI*-type locus was phylogenetically distinct from these previously characterized CDI systems. By contrast, the phylogenetic tree of CdiI (Fig 6) differed from those for CdiB, CdiA and CdiC and from bacterial species phylogeny.

Finally, we found no genes encoding orthologs of CdiI^FRM16^ in the other *Xenorhabdus* genomes. The closest CdiI^FRM16^ orthologs were encoded by genes in the *Photorhabdus* genus (*P*. *asymbiotica* ATCC43949, *P*. *heterorhabditis* VMG, *Photorhabdus temperata* M1021 and numerous strains of *P*. *luminescens*), in *Pseudomonas syringae* strains, and, more surprisingly, in a few strains of *Gilliamella apicola*, a gut symbiont of honey bees (Fig 6).

## Discussion

CDI systems may play a key role in competition strategies, by delivering toxins that kill neighbouring bacteria, thereby eliminating bacterial competitors. Known CDI systems are encoded by two different classes of genomic loci: those of the ‘‘*E*. *coli*-type” generally found in Enterobacteriaceae [27], and those of the ‘‘*Burkholderia*-type” found in the genera *Burkholderia*, *Cupriavidus*, *Ralstonia* [32, 33]. In this study, we used *Xenorhabdus* genomic resources to identify a new type of *cdi* locus, the *cdiBCAI*-type locus, in the *Xenorhabdus doucetiae* FRM16 genome. This locus is characterized by the presence of an additional ORF, located between *cdiB* and *cdiA* that we named *cdiC*, due to the orthology of its product with toxin-activating proteins C. Although the presence of such an accessory gene in *cdi* loci has been reported in some Gram-negative strains [43], it was never exhaustively investigated. A more exhaustive search of *cdiBCAI*-type loci in a bacterial database showed that the *cdiBCAI* type locus was frequently present in *Xenorhabdus genomes* (~ 30% of the sequenced genomes) and many environmental bacteria interacting with insects, plants, rhizosphere and soil. Previous studies have suggested that *cdi* loci probably undergo horizontal gene transfer (HGT) between bacteria, because they are often present in genomic and pathogenicity islands [26, 28]. The Xd_FRM16 *cdi* locus is the first example found to be located within an ICE, a class of mobile genetic elements known to mediate HGT [44, 45].

The CdiA toxin module of *X*. *doucetiae* FRM16, CdiA-CT^FRM16^, has many features in common with other described CdiA-CT. It is flanked at its C-terminal end by a VENN motif, and it is toxic when produced within the cells of closely or more distantly related bacteria [26]. CdiA-CT^FRM16^ is an Mg^2+^-dependent DNase, as shown for its ortholog CdiA-CT in *D*. *dadantii* strain 3937 [27]. Several other CdiA-CTs display nuclease activity *in vitro*, probably killing their target bacteria [27, 33, 46]. The CdiA-CT of *E*. *coli* EC869 has DNase activity, but its amino-acid sequence is very different from that of CdiA-CT^FRM16^ (30% identity) and its nuclease activity is Zn^2+^-dependent [47, 48]. This finding may reflect highly convergent evolution for the nuclease activity of CdiA-CT fragments.

We found that *cdiB*^FRM16^, *cdiC*^FRM16^, *cdiA*^FRM16^ and *cdiI*^FRM16^ were cotranscribed as an operon in both the exponential and the stationary phases of growth in LB culture. Moreover, we performed phylogenetic analysis, which displays that *cdiB*^FRM16^, *cdiC*^FRM16^ and *cdiA*^FRM16^ genes co-evolved. We therefore hypothesize that the product of the *cdiC* gene is involved in the functional CDI system. Due to the similarity of the CdiC protein with HlyC [36], CdiC^FRM16^ might play a role in activation of CDI through lysine acetylation. In our growth inhibition assay in *E*. *coli*, we did not observe any effect of the *cdiC*^FRM16^ expression on CdiA-CT^FRM16^ activity. Some authors suggest that CdiC activity may promote CdiA or CdiB association with membrane [49]. Indeed, CdiC^FRM16^ may play an important role in membrane-associated CDI functions, such as CdiBA biogenesis, CdiA target cell binding or toxin translocation through the cell envelope.

We identified and characterized the function of CdiI^FRM16^ in protecting the bacteria producing it from the toxic activity of CdiA-CT^FRM16^. Our phylogenetic analysis suggested that *cdiBCA* and *cdiI* were different evolutionary units. Ruhe and coworkers previously observed that, in many CDI toxin-immunity protein pairs, the CdiI proteins display much greater sequence diversity than the CdiA-CT toxins [26]. They suggest that *cdiI* evolution is rapid as long as CdiI maintains sufficient affinity for CdiA-CT to provide immunity [26]. Investigation of the genetic context of *cdiI*^*FRM16*^ orthologs in available complete genomes showed that they are either orphan genes or participate in orphan *cdiA-*CT-*cdiI* pairs (J-C. Ogier, unpublished data). These orphan *cdiI* genes or *cdiA-*CT-*cdiI* pairs may be horizontally exchanged between bacteria, potentially enabling bacteria to protect themselves against neighbouring bacteria that are producing and delivering CdiA-CT toxins. Interestingly, no *cdiI*^FRM16^ orthologs were found in *Xenorhabdus* genomes, and the closest *cdiI*^FRM16^orthologs, associated or unassociated with *cdiA-*CT, were mostly found in *Photorhabdus* strains, a genus having a life cycle similar to that of *Xenorhabdus*, including a major stage in the insect cadaver [22].

Several attempts for assessing role of the *in vivo* activity of the *cdiBCAI* locus were conducted between *X*. *doucetiae* FRM16 and strains of *Xenorhabdus* that naturally do not harbor any *cdi* loci. However, no clear effect could be detected (data not shown). This is likely due to the highly redundant arsenal of *Xenorhabdus* factors involved in antagonism between bacteria [4]. For example, a locus encoding a tail-phage bacteriocin is responsible for eliminating phylogenetically close bacteria from the insect cadaver [15].

In conclusion, *Xenorhabdus* genus is a pertinent resource for identifying genomic loci potentially involved in interbacterial competition systems, such as *cdi* loci. In order to determine the specificities of the *cdiBCAI*-type locus, further studies should be conducted.

## Supporting Information

S1 Table Bacterial strains, plasmids and primers used in the study (DOCX)

S2 Table List of strains used in the phylogenetic analysis and their accession numbers (XLSX)

S3 Table Genetic content of the genomic island (GI) inserted between the *fba* and *mltC* genes (XLSX)

S4 Table Taxonomic reports of orthologous sequences for CdiB^FRM16^, CdiC^FRM16^, CdiA^FRM16^ and CdiI^FRM16^ obtained by the BlastP method (protein-protein BLAST) (XLSX)

S1 Fig Comparison of the topologies of the phylogenetic trees of CdiB^FRM16^ (panel A), CdiC^FRM16^ (panel B) and CdiA^FRM16^ (panel C) in a limited set of bacterial species, as identified by BlastP (i.e. selection of 16 strains with complete *cdiBCAI* loci, covering species diversity). The phylogenetic trees were built by the maximum likelihood (ML) method, and branch support values (estimated by the aLRT(SH-like) method) are indicated at the nodes. The branch length scale bar below the phylogenetic tree reflects the number of amino-acid substitutions per site. The Xd_FRM16 sequences are highlighted in blue. For some taxa, orthologs are highlighted in red when the topology is not congruent with the species tree. The sequences from “*E*. *coli*-type” and “*Burkholderia*-type” *cdi* loci are used as outgroups, and are highlighted in bold. Accession numbers of the sequences are indicated in S2 Table.
(PPTX)
