# Supplementary material for: A New Member of the Growing Family of Contact-Dependent Growth Inhibition Systems in Xenorhabdus doucetiae
Source: PLoS One. 2016 Dec 1;11(12):e0167443. doi: 10.1371/journal.pone.0167443 (PMC5131962; doi:10.1371/journal.pone.0167443)
Supplement: S1 Table — (DOCX) [file pone.0167443.s001.docx]

**Table S1:** Bacterial strains, plasmids and primers used in the study

| **Strains** | **Relevant characteristic(s)** | **Reference or source** |
| --- | --- | --- |
| *Xenorhabdus doucetiae* FRM16 | Symbiont of nematode *Steinernema diaprepesi* isolated in Martinique (France) | [1] |
| *Xenorhabdus bovienii* CS03 | Symbiont of nematode *Steinernema weiseri* isolated in the Czech Republic | [2] |
| *E. coli* CopyCutter™ EPI400™ | F– *mcr*A Δ(*mrr-hsd*RMS-*mcr*BC) φ80d*lac*ZΔM15 Δ*lac*X74 *rec*A1 *end*A1 *ara*D139 Δ(*ara, leu*)7697 *gal*U *gal*K λ– *rps*L *nup*G *ton*A Δ*pcnB dhfr* | Epicentre |
| *E. coli* XL1-Blue MRF' | Δ(*mcrA*)*183* Δ(*mcrCB-hsdSMR-mrr*)*173 endA1 supE44 thi-1 recA1* gyrA96 relA1 lac *[F′* proAB lacI^q^*ZΔ*M15 *Tn*10 *(Tet^r^)]* | Stratagene |
| *E. coli* BL21 | F– *mcr*A Δ(*mrr-hsd*RMS-*mcr*BC) φ80d*lac*ZΔM15 Δ*lac*X74 *rec*A1 *end*A1 *ara*D139 Δ(*ara, leu*)7697 *gal*U *gal*K λ– *rps*L *nup*G *ton*A Δ*pcnB dhfr* | Stratagene |
| *E. coli* WM3064 (derived from B2155, which is auxotrophic for DAP and used for conjugation experiments) | *thrB1004 pro thi rpsL hsdS lacZΔM15 RP4-1360 Δ(araBAD)567 ΔdapA1341::[erm pir]* | [3] |
| **Plasmids** | **Description** | **Reference or source** |
| pGJ907 (=P*tet*-MCS) | Cloning vector, P*_tet_* promoter, tet^R^, Km^R^ | [4] |
| pGJ907_*cdiA*-CT^FRM16^ | *cdiA-* CT^FRM16^ from Xd_FRM16 inserted between EcoRI and SalI in pGJ907 cloning vector, under the control of the P*tet* promoter, Km^R^ | This study |
| pUC18 | Cloning vector, P*lac* promoter, Amp^R^ | Stratagene |
| pUC18_XDD1_1118 | XDD1_1118 gene from Xd_FRM16 inserted between the *Eco*RI and *Sal*I sites in the pUC18 cloning vector, under the control of the P*lac* promoter, Amp^R^ | This study |
| pUC18_ XDD1_1120 | XDD1_1120 gene from Xd_FRM16 inserted between the *Eco*RI and *Sal*I sites in the pUC18 cloning vector, under P*lac* promoter control, Amp^R^ | This study |
| pET28b | Cloning vector, T7 promoter, Km^R^ | Novagen |
| pET28b_ *cdiA*-CT^FRM16^_ XDD1_1120 | *cdiA-* CT^FRM16^, and XDD1_1120 from Xd_FRM16 inserted between the *Eco*RI and *Sal*I sites in the pET28b cloning vector, under the control of the PT7 promoter, Km^R^ | This study |
| pET28b_ XD1120 | XDD1_1120 from Xd_FRM16 inserted between the *Eco*RI and *Sal*I sites in the pET28b cloning vector, under the control of the PT7 promoter, Km^R^ | This study |
| **Oligonucleotides** | **Sequences (5’ →3’)** | **Reference or source** |
| **Genetics** | | |
| F_EcorI_Cter_tpsA_III | gcgcgaattcAAAGAGGTGCTGAACGATGACAATTGCAGGAGGTCTGGCGGCAGGGCTG | This study |
| R_sal1_tpsA_III | gcgcgtcgacAGATGATTCTGTTGTTCCAAGG | This study |
| XD1120_EcoR1_F | GCGCGAATTCAATCTTTGTGGGGCTGTATGTC | This study |
| XD1120_sal1_R | gcgcgtcgacGCTAACAGCTAAAGGGAAGCTC | This study |
| XD1118_EcoR1_F | GCGCGAATTCATAACGGCCATGTCACTTTCAG | This study |
| XD1118_sal1_R | gcgcgtcgacTCAGACACGACCATCAACATCT | This study |
| XD_1120_sal1R_bis | gcgcgtcgacAAGCAACATTAAACTACCGTTACAT | This study |
| **RT-PCR** | | |
| 16SP1 | GAAGAGTTTGATCATGGCTC | [5] |
| 16SP2 | AAGGAGGTGATCCAGCCGCA | [5] |
| 1_F | ACTGAGTGACCTGCTTTATCTCTC | This study |
| 1_R | ATATTTAGCATCCCCGTTCAGTTC | This study |
| 2_F | ACGGTTTGATGAAGAGACTGAAC | This study |
| 2_R | GGACACACTGATAAGGAAAAATGT | This study |
| 3_F | CCAGATGAGAAGGAAAAAGTCAGTA | This study |
| 3_R | GCTTTAAGGCTTTCTGGTATTTGTC | This study |
| 4_F | TAGGAGAGCAACCCGAAGTATC | This study |
| 4_R | GTTTAATACCCCCTCTCGGAAG | This study |
| 5_F | GCACCGGAGCAGGATTTAAGTCA | This study |
| 5_R | CAGGTTACCATGTTCATCCGTGC | This study |
| 6_F | TGGACATCACTACCGCAGAAGAG | This study |
| 6_R | TGCCAGTAATCCCTGCCGGTT | This study |
| 7_F | CACGGTGAAGCTCAATGCAGAGA | This study |
| 7_R | TCCCATCCTTCGCCAATGTTAGA | This study |

1. Fischer Le Saux M, Mauleon H, Constant P, Brunel B, Boemare N. PCR-ribotyping of *Xenorhabdus* and *Photorhabdus* isolates from the Caribbean region in relation to the taxonomy and geographic distribution of their nematode hosts. Appl Environ Microbiol. 1998; 64(11):4246-54. PubMed PMID: ISI:000076694200018.

2. Bisch G, Pages S, McMullen JG, Stock SP, Duvic B, Givaudan A, et al. *Xenorhabdus bovienii* CS03, the bacterial symbiont of the entomopathogenic nematode *Steinernema weiseri*, is a non-virulent strain against lepidopteran insects. J Invertebr Pathol. 2015; 124:15-22. PubMed PMID: ISI:000348250600003.

3. Paulick A, Koerdt A, Lassak J, Huntley S, Wilms I, Narberhaus F, et al. Two different stator systems drive a single polar flagellum in *Shewanella oneidensis* MR-1. Mol Microbiol. 2009; 71(4):836-50. PubMed PMID: ISI:000263137400004.

4. Jubelin G, Lanois A, Severac D, Rialle S, Longin C, Gaudriault S, et al. FliZ Is a Global Regulatory Protein Affecting the Expression of Flagellar and Virulence Genes in Individual *Xenorhabdus nematophila* Bacterial Cells. PLoS Genet. 2013; 9(10). PubMed PMID: ISI:000330367200076.

5. Tailliez P, Pages S, Ginibre N, Boemare N. New insight into diversity in the genus *Xenorhabdus*, including the description of ten novel species. Int J Syst Evol Microbiol. 2006; 56:2805-18. PubMed PMID: ISI:000243087100014.
