# Supplementary material for: A New Member of the Growing Family of Contact-Dependent Growth Inhibition Systems in Xenorhabdus doucetiae
Source: PLoS One. 2016 Dec 1;11(12):e0167443. doi: 10.1371/journal.pone.0167443 (PMC5131962; doi:10.1371/journal.pone.0167443)
Supplement: S1 Fig — Comparison of the topologies of the phylogenetic trees of CdiBFRM16 (panel A), CdiCFRM16 (panel B) and CdiAFRM16 (panel C) in a limited set of bacterial species, as identified by BlastP (i.e. selection of 16 strains with complete cdiBCAI loci, covering species diversity). The phylogenetic trees were built by the maximum likelihood (ML) method, and branch support values (estimated by the aLRT(SH-like) method) are indicated at the nodes. The branch length scale bar below the phylogenetic tree reflects the number of amino-acid substitutions per site. The Xd_FRM16 sequences are highlighted in blue. For some taxa, orthologs are highlighted in red when the topology is not congruent with the species tree. The sequences from “E. coli-type” and “Burkholderia-type” cdi loci are used as outgroups, and are highlighted in bold. Accession numbers of the sequences are indicated in S2 Table. (PPTX) [file pone.0167443.s005.pptx]

## Slide 1
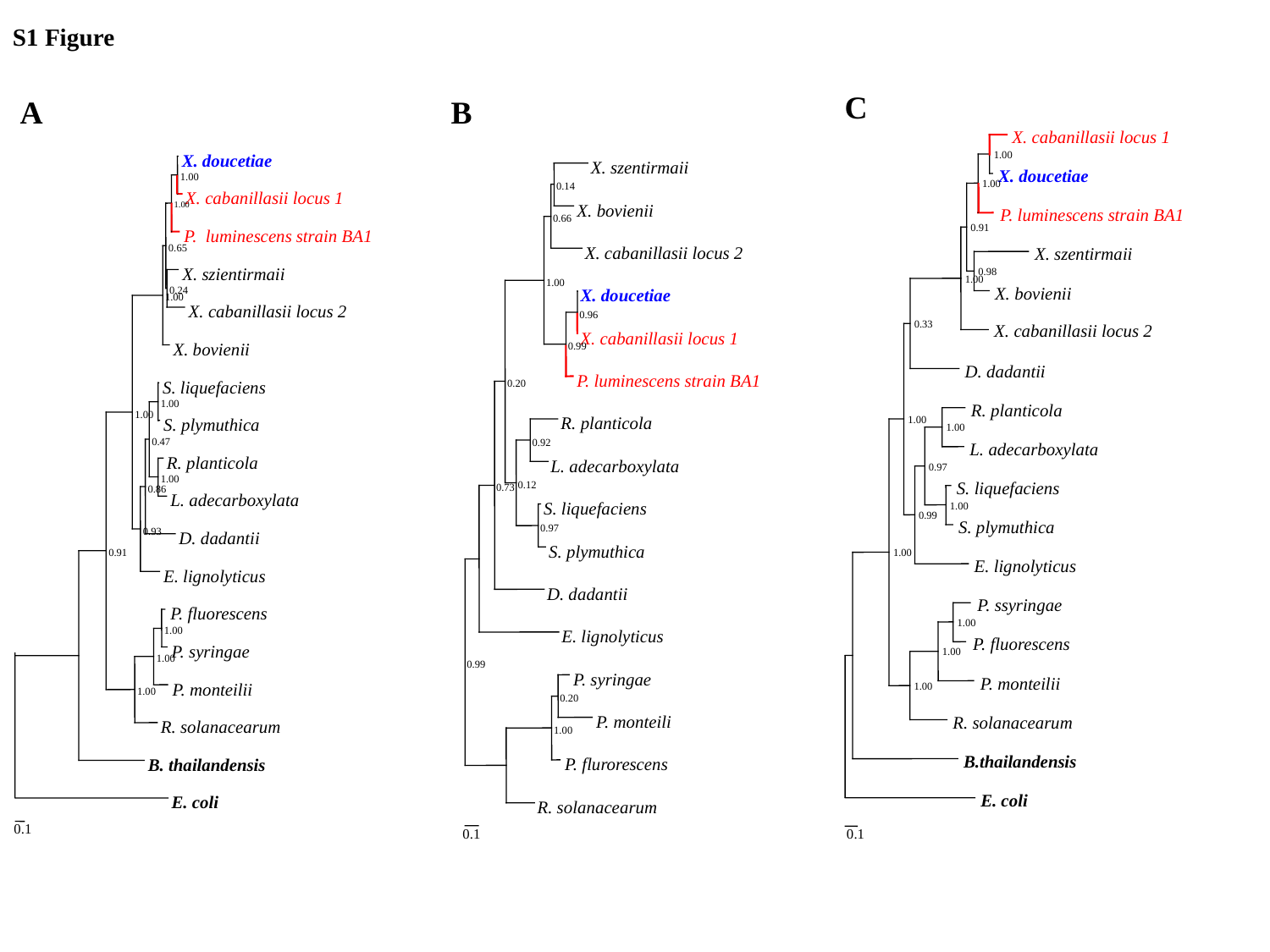

S1 Figure
C
A
B
X. cabanillasii locus 1
1.00
X. doucetiae
X. szentirmaii
X. doucetiae
1.00
1.00
0.14
X. cabanillasii locus 1
1.00
X. bovienii
P. luminescens strain BA1
0.66
0.91
P. luminescens strain BA1
0.65
X. cabanillasii locus 2
X. szentirmaii
X. szientirmaii
0.98
1.00
1.00
X. bovienii
0.24
X. doucetiae
1.00
X. cabanillasii locus 2
0.96
0.33
X. cabanillasii locus 2
X. cabanillasii locus 1
X. bovienii
0.99
D. dadantii
P. luminescens strain BA1
S. liquefaciens
0.20
1.00
R. planticola
1.00
R. planticola
1.00
S. plymuthica
1.00
0.47
0.92
L. adecarboxylata
R. planticola
L. adecarboxylata
0.97
1.00
S. liquefaciens
0.12
0.73
0.86
L. adecarboxylata
S. liquefaciens
1.00
0.99
S. plymuthica
0.97
0.93
D. dadantii
S. plymuthica
0.91
1.00
E. lignolyticus
E. lignolyticus
D. dadantii
P. ssyringae
P. fluorescens
1.00
1.00
E. lignolyticus
P. fluorescens
P. syringae
1.00
1.00
0.99
P. syringae
P. monteilii
P. monteilii
1.00
1.00
0.20
P. monteili
R. solanacearum
R. solanacearum
1.00
B.thailandensis
P. flurorescens
B. thailandensis
E. coli
E. coli
R. solanacearum
0.1
0.1
0.1
